# A Modified Kulka Micromethod for the Rapid and Safe Analysis of Fructose and 1-Deoxy-d-xylulose-5-phosphate

**DOI:** 10.3390/metabo8040077

**Published:** 2018-11-08

**Authors:** Shreya Shaw, Robin Ghosh

**Affiliations:** Department of Bioenergetics, Institute of Biomaterials and Biomolecular Systems, University of Stuttgart, Pfaffenwaldring 57, D-70569 Stuttgart, Germany; st154798@stud.uni-stuttgart.de

**Keywords:** fructose assay, 1-deoxy-d-xylulose-5-phosphate, resorcinol reagent, micromethod, systems biology, enzyme assay

## Abstract

The Kulka resorcinol assay (Kulka, R.G., *Biochemistry* 1956, *63*, 542–548) for ketoses has been widely used in the literature but suffers from two major disadvantages: (a) it employs large amounts of potentially harmful reagents for a general biology laboratory environment; and (b) in its original formulation, it is unsuited for modern high-throughput applications. Here, we have developed a modified Kulka assay, which contains a safer formulation, employing approx. 5.4 M HCl in 250 µL aliquots, and is suitable for use in high-throughput systems biology or enzymatic applications. The modified assay has been tested extensively for the measurement of two ketoses—fructose (a common substrate in cell growth experiments) and 1-deoxy-d-xylulose-5-phosphate (DXP), the product of the DXP-synthase reaction—which until now has only been assayable using time-consuming chromatographic methods or radioactivity. The Kulka microassay has a sensitivity of 0–250 nmol fructose or 0–500 nmol DXP. The assay is suitable for monitoring the consumption of fructose in bacterial growth experiments but is too insensitive to be used directly for the measurement of DXP in in vitro enzyme assays. However, we show that after concentration of the DXP-enzyme mix by butanol extraction, the Kulka resorcinol method can be used for enzyme assays.

## 1. Introduction

The determination of sugars in biological assays is a common task in many areas of biochemistry, fermentation, and more recently, systems biology. The most sophisticated methods for sugar determination usually involve high-performance liquid chromatography (HPLC) techniques [1,2,3,4,5,6], which allow both sugar identification as well as their precise quantitation. However, HPLC techniques are inherently slow, and also usually require deproteinization of biological samples prior to analysis. An alternative is gas-liquid chromatography [3,4,7], which though precise, requires derivatization procedures, as well as requiring long run times, and is thus cumbersome for a large number of samples.

For studies requiring the rapid analysis of a large number of samples, e.g., in areas of enzyme kinetics, fermentation, or systems biology, colorimetric methods involving usually absorbance or fluorescence measurements are still of great utility due to their potential for high-throughput as well as being suitable for automated techniques. Many methods for the colorimetric measurements of furanoses and pyranoses exist, some of which involve direct measurement of extracted samples [8,9,10,11,12,13,14,15], whereas others require prior rapid purification by thin-layer chromatography [16,17,18,19]. However, many of these methods are today, in view of laboratory safety regulations, only of historical importance, as they usually involve large quantities of acids or reagents with significant potential as carcinogens. Nevertheless, we show here that, at least for the rapid determination of furanoses, in particular here, d-fructose, d-xylulose, and 1-deoxy-d-xylulose-5-phosphate (DXP), the historically important Kulka method [10] can be modified as a micromethod, suitable for the determination of nanomolar amounts of fructose or DXP. The method can be adapted for the rapid handling of samples using modern techniques, and also provides a much higher level of biological and laboratory safety than the original method. Nevertheless, the modified Kulka method retains its well-known reliability for furanose determination.

**Figure 1:**
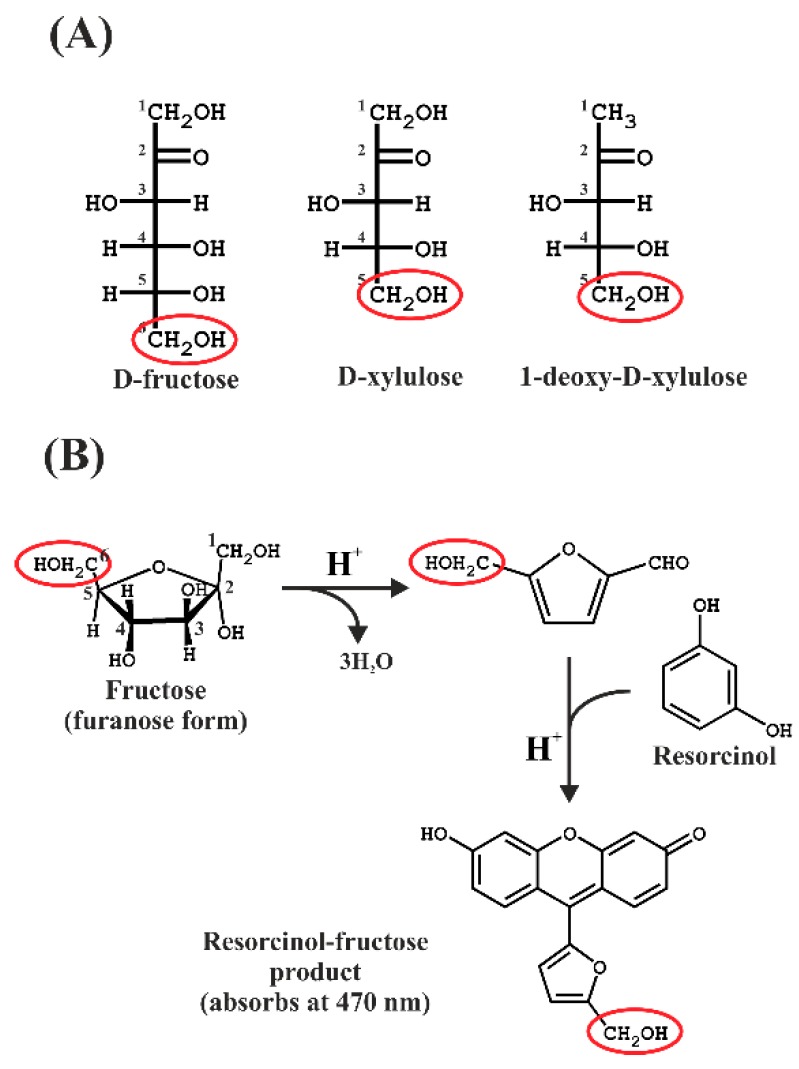
(**A**) Fisher projections of the sugars used in this study. The hydroxymethyl groups remaining after the furfural formation are indicated by red ellipses. (**B**) Reaction scheme showing the formation of the furfural intermediate, which then reacts with resorcinol in the presence of hot concentrated acid.

The Kulka method [10], an adaptation of the resorcinol method described by Bacon and Bell [11], involves the ferric ammonium sulphate-mediated oxidation of furanose in concentrated HCl acid to form the furfural product from furanose, followed by reaction with the dye resorcinol to form a colored product, which can then be quantitated by absorption spectroscopy. The basic reaction scheme is shown in Figure 1.

In the original Kulka procedure [10], the acid step was performed using concentrated HCl (approximately 11.8 M), which must be heated for 30 min at 80 °C in order to form the furfural product. The high HCl concentration not only prohibits the use of normal automatic pipettes but poses a considerable danger to the experimentalist as well as the laboratory environment in case of an accident.

In our study, we show that levels of acid as low as 5.38 M HCl (which can be handled with a normal automatic pipette) are still sufficient to allow efficient furfural formation from furanose, and that once formed, the product can be diluted with water to yield a final acid concentration of about 1.2 M, which presents only minimal risk to the user and laboratory environment. Nevertheless, the new modified Kulka assay is able to reliably determine fructose and DXP in the micromolar range. The final protocol is described in detail in the Materials and Methods section.

## 2. Results

### 2.1. General Considerations

Although, in the original Kulka method, the whole procedure was carried out in concentrated HCl [10], we show below that the initial formation of furfural is also efficiently performed in the presence of approximately 6 M HCl (the final [HCl] in the assay is 5.38 M), here used in a final volume of only 225–245 µL volumes. As mentioned above, this lower concentration of acid can also be pipetted with automatic pipettes commonly found in most laboratories. In addition, since 6 M HCl is a constant boiling mixture, the Fe(III)-HCl reagent (solution A) is quite stable at room temperature in a closed bottle. We reasoned that, once formed, the furfural-resorcinol product should be stable in water, allowing us to dilute the reaction mixture further to yield a final acid concentration of about 1.2 M, which can be safely handled in the laboratory. However, as we show below, this last dilution leads to some precipitation of non-reacted furfural product, necessitating a centrifugation step prior to measurement, as well as an absorption measurement at two wavelengths to ensure reliable calibration. These latter steps contribute only minimally to the total procedural time.

### 2.2. Spectral Analysis of the Fructose- and DXP-Resorcinol Reaction Mixtures

**Figure 2:**
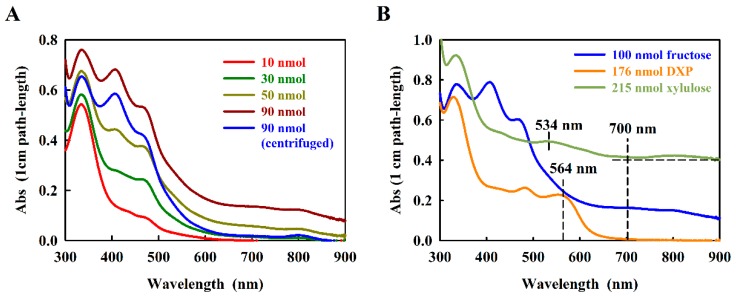
Absorption spectra of the resorcinol adducts with (**A**) d-fructose, and (**B**) d-xylulose and DXP. Concentrations of sugar are indicated. The relevant measurement wavelengths are indicated with dashed vertical lines. The horizontal dashed line shows the baseline value used to calculate the d-xylulose baseline-corrected spectra.

In the original work, Kulka [10] showed that the fructose-furfural product reacted with resorcinol to produce a final product exhibiting two characteristic absorption maxima, which were assigned to 415 nm and 480 nm, respectively. When we re-measured the absorption spectrum of the fructose-resorcinol product (here using a modern scanning spectrophotometer) we confirmed the general form of the spectrum, but in 1.2 M HCl, three absorption maxima at 470 nm, 417 nm, and 333 nm, respectively, were observed (Figure 2A).

The absorption spectrum of the fructose-resorcinol product was complex (Figure 2A), containing so far undocumented very weak absorption bands at about 700 nm and 820 nm. We noted that for higher amounts of fructose (at about 50 nmol fructose), the turbidity of the product rose significantly in the 1.2 M HCl measuring solution, even though the reaction product absorption at 470 nm still increased with the amount of fructose assayed. This problem was eliminated by a 5 min centrifugation step prior to measurement (see Figure 2A). To eliminate the very small baseline contribution at 700 nm, the absorption difference (A_470_–A_700_) proved to be reliable for estimating the concentration dependence.

We have not performed an exhaustive analysis of furanoses, as many of these were reported in the original Kulka [10] publication.

However, in a second step of the work, we chose to focus on the possible use of resorcinol to assay DXP, which to our knowledge has never been reported. Kulka [10] reported that the related furanose, d-xylulose, is still able to react with resorcinol, yielding a green product showing about one tenth of the absorption observed with fructose. The xylulose-furfural derivative still contains a single aldehyde group, derived from the C-1-hydroxymethyl moiety (Figure 1A), and is thus able to form a limited resorcinol product, although the final structure has not been reported. In DXP, the C-1-hydroxymethyl group is absent, which would seem to preclude a reaction with resorcinol. In initial trials, we found that, surprisingly, DXP reacts well with resorcinol, forming a red product with a characteristic absorption at 564 nm (Figure 2B). The absorption intensity of the DXP-resorcinol product is about 5-fold higher than that of the baseline-corrected d-xylulose product at its corresponding characteristic absorption maximum (534 nm, Figure 2B). Although we have not determined the structure of the DXP-resorcinol product, we assumed that the acid step was sufficient to remove the phosphate group by hydrolysis, thus allowing the resulting C-4 hydroxymethyl group to become available to react with the C-2 carbonyl, allowing furfural formation. However, the subsequent reaction with resorcinol must have been unusual, since the reactive C-1 hydroxymethyl group was replaced with an unreactive methyl moiety. Nevertheless, the absorption of the DXP-resorcinol product at 564 nm was distinct, and as an added bonus, showed almost negligible turbidity in 1.2 M HCl.

### 2.3. Calibration Guidelines for Fructose and DXP Using the Modified Kulka Method

#### 2.3.1. Calibration with Fructose

**Figure 3:**
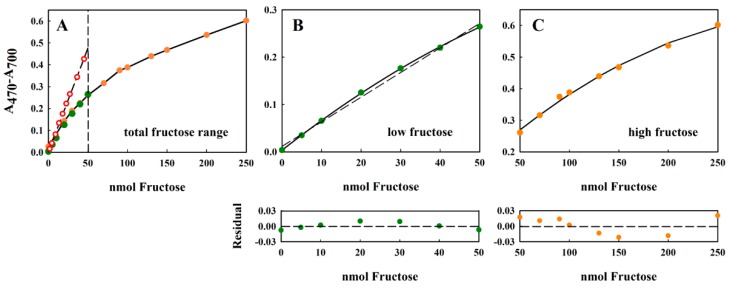
Standard curve of fructose showing good overlap between the absorption values measured with the 2.5 mM and 10 mM stock solutions. All measurements were performed in triplicate, and the average value is indicated. The error of the measurement was usually about 2% (data not shown). The separated ranges, indicated by the dashed line in panel (**A**) 0–50 nmol fructose and 50–250 nmol fructose, have been extracted from (**A**) and plotted separately in panels (**B**,**C**), respectively. For the data in both panels B and C, the solid lines correspond to a polynomial regression to the equation y = y_o_ + ax + bx^2^, whereas the dashed line shown in panel B corresponds to a linear regression to the equation: y = y_o_ + ax. For the 0–50 nmol fructose data set, the polynomial constants were: y_o_ = 0.0033 ± 0.0013; a = 0.0066 ± 0.0001; b = −2.728 × 10^–5^ ± 2.67 × 10^−6^. For the linear fit to the same data, the fitted constants were: y_o_ = 0.0114 ± 0.0047; a = 0.0052 ± 0.0002. For the 50–250 nmol fructose data set, the polynomial constants were: y_o_ = 0.0629 ± 0.136; a = 0.004 ± 0.0004; and b = −7.71 × 10^−6^ ± 2.73 × 10^−8^. The quality of the fit has been shown using the residual plot (lower panels under (**B**,**C**)), defined as {(A_470_–A_700_)_exptl_ − (A_470_–A_700_)_calc_}, where (A_470_−A_700_)_calc_ has been calculated from the fitted equation. For the 0–50 nmol fructose range, only the residual derived from the linear fit is shown. In panel (**A**), the original Kulka data [10], recalculated to include the four-fold dilution in our assay, is also shown (red circles, dashed line).

In the original Kulka method [10], a calibration curve of 0–100 µg/mL sample (fructose) solution was shown. The highest value corresponded to a fructose concentration of 555 µM. In this measurement range, the absorption maximum at 480 nm (the measuring wavelength used by Kulka [10]) appeared to vary almost linearly with the fructose concentration. Our own measurements, using our modified Kulka procedure, shows that the attainable dynamic range was much higher than that originally reported in the original publication. As the data in Figure 3 shows, the modified Kulka method could reliably measure concentrations of 0–250 nmol fructose, present in the 20 µL aqueous sample (corresponding to 12.5 mM fructose) added to the resorcinol-HCl reagent.

The assay could tolerate up to a 20 µL sample without detrimentally diluting the acid. This range corresponded to the concentrations 0–12.5 mM or 0–2250 µg/mL fructose. The non-linearity of the large dynamic range (Figure 3A) could be addressed easily by separating the curves into “low” (0–50 nmol fructose (Figure 3B)) and “high” (50–250 nmol fructose (Figure 3C)) measurement regions. In both cases, the curves could be well-fitted using (different) second-order polynomials of the form y = y_0_ + ax + bx^2^, where x and y are the concentration of fructose and the difference absorption value (A_470_–A_700_), respectively, and y_0_, a, and b are constants to be determined by curve-fitting procedures (see Figure 3, legend). In the absence of access to curve-fitting software, the concentration dependence of the lower range was almost linear (indicated by the low sum of squares Σ(y_expt_ − y_calc_)^2^ = 0.172), and the values thus obtained by linear regression showed only a small deviation from those obtained after polynomial fitting (Figure 3B, residual panel). Although the residual value is a commonly used direction vector for regression algorithms, and is a sufficient fitting criterion for precise measurements; more extensive tests of linearity can be found in the review of Araujo [20].

We also examined the stability of the final reaction product. We found, after incubation of the centrifuged and diluted reaction product for a series of time points (data not shown), that the reaction stayed completely stable for at least 2 h after the reaction, provided that the final cocktail was not mixed. Regular agitation of the final reaction cocktail led to significant loss of the A_470_ product, presumably due to an oxidative side-reaction. However, in the absence of mixing, the A_470_ product decreased by only about 10% after 6 h at room temperature. We emphasize, however, that performance of the reaction as described in the Methods section is extremely reliable and reproducible.

Comparison of the results obtained here for the modified method with those originally reported by Kulka [10] (Figure 3A, red circles, dashed line) show that the original Kulka method was more sensitive at lower concentrations of fructose (note that the A_480_ values reported by Kulka [10] have been reduced 4.08-fold to take the different dilutions employed by the two methods into account). However, our modified method shows a higher dynamic range, thus making high fructose concentrations accessible to the assay. The reason for the discrepancy between the two procedures may be due to the different reaction conditions used.

#### 2.3.2. Effects of Interfering Substances upon the Modified Kulka Assay

Biochemical reaction cocktails usually employed in enzyme assays contain a variety of substances that may interfere with the modified Kulka assay. We have analyzed the effects of several commonly used buffers and salts at concentrations typical for their use in enzyme assays, and the results are shown in Table 1.

The effect of interference was quantified by measuring a single, low concentration of fructose (20 nmol) in the presence of the interfering reagent. In the event, commonly used buffers, such as TrisHCl, Na-phosphate, 4-(2-hydroxethyl)-1-piperazine-ethane sulphonic acid (HEPES), and 3-(N-morpholino)propane sulphonic acid) (MOPS), as well as NaCl, each sampled (20 µL sample) from a 100 mM stock solution, had either negligible or only very minor effects upon the assay. No effect of MgCl_2_ (taken from a 10 mM stock solution) was also observed.

The effects of proteins on the assay were more complicated. We observed that large concentrations (mg amounts) of the model proteins bovine serum albumin (BSA) and lysozyme in the solutions to be assayed have a significant effect upon the modified Kulka assay (increasing the A_470_–A_700_) by up to 165% (for a protein sample concentration of 5 mg/mL) of the control measured in the absence of protein). However, lower protein concentrations (100 µg/mL), typical for the stabilization of enzymes, had only a small (increase to 120%) effect upon the assay (see Table 1). It is known that both BSA (which contains 17 disulphide bridges) and lysozyme retain significant tertiary structure upon acid denaturation [21], which still allows binding of aromatic substances (such as resorcinol and its adducts) to exposed hydrophobic pockets, so the effects at the higher concentrations are to be expected. At the lower concentrations, the molar ratio of BSA/resorcinol (1/152 (mol/mol)) was too low to be significant. Nevertheless, proteins are often present in biochemical assays at higher concentrations, which might limit the applicability of the modified assay. However, when we performed an initial deproteinization of the protein-containing sample by preincubating with 3.4% (*w*/*v*) trichloroacetic acid (TCA) followed by a 20 min centrifugation step to remove the protein pellet (see Materials and Method for details), the values obtained for 20 nmol fructose were very close to those obtained in the absence of both protein and TCA. In control assays we could demonstrate that within the range 3.4–5% TCA (which is sufficient for deproteinization), furfural development was only weakly dependent upon the amount of TCA added (Table 1). We were also able to demonstrate that the form of the fructose calibration curve was unchanged compared to the data shown in Figure 3A. However, routinely, the fructose calibration curve should be performed including the TCA precipitation step to be used in the experiment.

Finally, we have examined the effects of glucose upon the fructose concentrations obtained with the modified assay. At equimolar concentrations of fructose and glucose (20 nmol, respectively), the absorption values A_470_–A_700_ were increased by only about 1–3% (Table 1) which is consistent with data originally reported by Kulka [10]. However, when the molar ratio of glucose/fructose was set to 110, the A_470_–A_700_ values were increased by about 90%. A control assay containing high [glucose] alone also showed a high A_470_–A_700_ value (see Table 1). We have not examined these effects systematically, since our results indicate that fructose measurements in the presence of about 100-fold higher concentrations of glucose will be unreliable and should be determined by another method. Nevertheless, in situations where the glucose and fructose concentrations are comparable (for example, in a sugar isomerase reaction) the Kulka method will be reliable.

Finally, we could show that the phosphorylated forms of fructose, fructose-6-phosphate (F6P), and fructose-1,6-diphosphate (F1,6diP) could also be determined with the modified Kulka assay, although both sugar phosphates yielded lower A_470_–A_700_ values (F6P, 74% of the control (fructose); F1,6diP; 87.5% of the control) compared to those of non-phosphorylated fructose when incubated for 40 min at 80 °C. However, extension of the 80 °C reaction step to 120 min increased the value for F1,6diP to that of the control, and also increased the value for F6P. The latter value was about 81% of the control measured after 40 min.

#### 2.3.3. Application of the Modified Kulka Assay to Determine the Consumption of Fructose in a Bacterial Growth Experiment

To demonstrate a typical application of the modified Kulka method developed here, we measured the consumption of fructose in a bacterial growth experiment. We have shown previously [22,23] that the purple bacterium *Rhodospirillum rubrum* will grow semi-aerobically in the dark in shake flasks in the presence of the carbon sources succinate and fructose. In this experiment, succinate rapidly attained a steady-state level, whereas fructose was consumed continuously throughout the major part of the growth experiment. In the final phase of the growth experiment, the fermentation product acetate, produced from fructose metabolism, was then further metabolized by the organism until the end of the growth phase [23].

**Figure 4:**
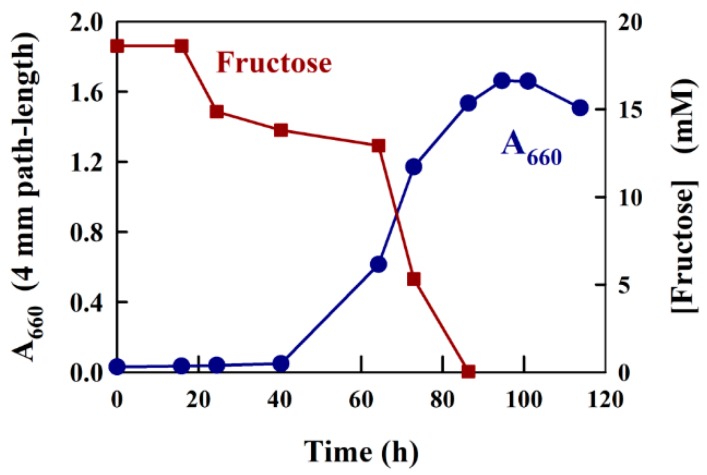
Growth curve data and fructose measurement for *R. rubrum* growing semi-aerobically on fructose/succinate M2SF medium in a shake flask at 30 °C (dark conditions). The cell turbidity (A_660_) was measured at 660 nm, which contained no contribution from photosynthetic pigments.

A typical growth experiment using the medium, M2SF [22], a modification of Sistrom medium A [24], which contains both 20 mM succinate and 8 mM ammonium chloride as C- and N-sources, respectively, as well as a large number of trace elements [24], with the addition of 18 mM fructose as a second carbon source, is shown in Figure 4. Prior to the determination of fructose in this experiment, we tested whether Sistrom medium A reacted with the modified Kulka reagent. During the test, no significant reaction was observed. For the experiment shown in Figure 4, a slightly aged (a stationary phase culture kept at 6 °C for two weeks) *R. rubrum* culture was employed as an inoculum. As expected, the A_660_ profile indicated that the culture showed an approximately 20 h lag phase, followed by normal growth (the generation time is about 3.5 h for semi-aerobic growth [22]) for about 60 h, finally reaching a stationary phase with A_660_ (4 mm path-length) values of approximately 1.6. For the determination of fructose in the medium supernatant, we precipitated 1 mL culture samples using 4.6% (*w*/*v*) TCA (see Materials and Methods) and then assayed the fructose present in a 10 or 20 µL aliquot. The determination of fructose consumption with the modified Kulka assay yielded satisfying results, completely consistent with previous experiments where HPLC determination of fructose was employed [23]. Thus, following the initial 20 h lag phase, where no fructose consumption was observed, the early, aerobic growth phase (up to an A_660_ of about 0.5) showed a low rate of fructose consumption. We have shown previously, that under aerobic conditions, succinate is used preferentially before fructose [23]. However, when the cells enter the semi-aerobic growth phase (above an A_660_ of about 0.5), the pathways of anaerobic metabolism are induced, which lead to rapid fructose consumption and exhaustion (at about 82 h). In the final growth phase, the acetate formed by fructose catabolism induces the ethylmalonyl-CoA pathway [25], allowing acetate to be utilized as a growth substrate [23].

#### 2.3.4. The Determination of DXP Using the Modified Kulka Assay

**Figure 5:**
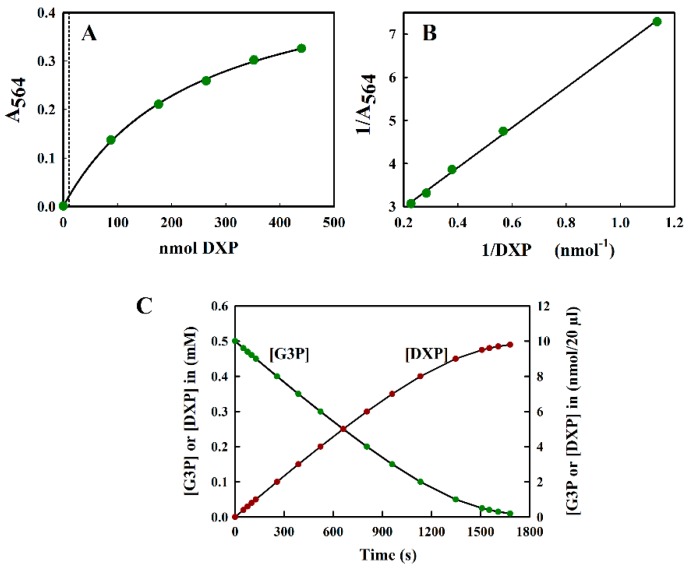
(**A**) The standard curve of DXP measured at 564 nm. All measurements were performed in triplicate, and the average value is indicated. The curve could be fitted to the hyperbolic equation y = ax/(b + x), where the fitted constants were: a = 0.507 ± 0.0124 and b = 244.323 ± 12.946. The dotted line indicates the upper limit (10 nmol) of the region relevant for the DXS assay shown in (**C**). (**B**) The inverse plot of the data shown in (**A**). (**C**) The theoretical progress curve of the DXS reaction, with pyruvate and G3P as substrates. The pyruvate concentration was assumed to be kept at a saturating level, whilst the G3P concentration steadily decreases as DXP is formed.

The reaction of DXP with resorcinol was measured at 564 nm. Since the spectral analysis showed no turbidity due to the reaction product, we found it unnecessary to perform a control measurement at 700 nm (see Figure 2B). The calibration curve A_564_ versus nmol DXP was curved (Figure 5A) and, unlike the fructose calibration curve, could be fitted well using a hyperbolic equation. This naturally led to the use of an inverse plot for routine calibration purposes (Figure 5B). Absorbance values were stable for up to 1 h, after which they steadily decreased to 77% of the initial value after 2 h.

The sensitivity of the modified Kulka assay for DXP was limited to about 2 mM (i.e., 40 nmol DXP/20 µL sample), which was more than sufficient for the determination of DXP in situations where DXP was being overproduced in cells for production purposes.

#### 2.3.5. Application to the Assay of DXP Synthase (DXS)

To illustrate the problems of DXP determination in a typical DXS assay, we simulated a theoretical progress curve for the consumption of substrate (glyceraldehyde-3-phosphate (G3P)) and the production of product, DXP, using the kinetic constants reported by Eubanks and Poulter [26] for the DXS enzyme from *Rhodobacter capsulatus* using a typical value of saturating [G3P] (0.5 mM) as a starting point. In this simulation, we assumed, for simplicity, that the reaction goes to completion. In reality, an equilibrium [DXP] was reached, significantly below the maximal value shown in Figure 5C. We also assumed that the reaction was performed in a reaction volume of 200 µL, which could be accommodated into the well of a 96-well plate. Figure 5C indicates that the maximal amount of DXP expected was not more than 10 nmol/20 µL aliquot (the largest volume tolerated in the modified Kulka assay), taken directly from the assay mix, which was too low to be estimated reliably by our method (see Figure 5A).

To solve the sensitivity problem, we examined two methods for increasing the effective sugar phosphate concentration. First, in a control experiment, we attempted to precipitate the sugar phosphate, fructose-6-phosphate (F6P), with BaCl_2_, since Ba-phosphate salts are water-insoluble [27,28]. We found that an [F6P] of 0.2–0.5 mM could be precipitated via the addition of a 100-fold higher concentration of BaCl_2_ in the presence of 50% ethanol. However, for higher F6P concentrations, even a 10-fold molar excess of BaCl_2_ was found to be sufficient for precipitation. We enhanced the efficiency of precipitation by solubilizing F6P in 5 mM Na-phosphate buffer pH 8.0. The buffer not only ensured that the F6P phosphate moiety was ionized, but also acted as a phosphate carrier and, after centrifugation, yielded a clearly visibly white precipitate after the addition of BaCl_2_ to the F6P solution. BaCl_2_ and 5 mM Na-phosphate were shown to have no effects upon the assay. The Ba^2+^-F6P precipitate went into solution upon the addition of ferric ammonium sulphate-HCl (FAS-HCl) reagent. The resuspended F6P samples yielded A_564_ values that were 90–100% of the control values obtained for 20–50 nmol F6P samples dissolved in H_2_O.

The slightly low value for F6P obtained using the Ba^2+^-precipitation method may have been due to the fact that about 1% of the phosphate moiety was in the singly charged form at pH 8.0 and may precipitate less effectively with Ba^2+^. Unfortunately, the precipitation of DXP via ethanolic BaCl_2_ was poor and unreliable, yielding values as low as 60% of the DXP control samples.

Thus, as an alternative, we attempted to concentrate the aqueous phase of large aliquots via repeated extraction with *n*-butanol [29]. In this procedure, the charged DXP should remain in the aqueous phase, thereby increasing its concentration, and hence accessibility in the modified Kulka assay. During the experiment, we were able to achieve a 4-fold reduction in the volume of the 100 µL samples, containing 0.05–0.5 nmol DXP, by extracting twice with 200 µL *n*-butanol. Smaller volumes of *n*-butanol were used if small reductions in volume were desired. The A_564_ values obtained for the extracted samples were between 85–100% of the DXP control samples. Routinely, for the determination of an unknown dilute [DXP], a calibration curve obtained from initially dilute, *n*-butanol-extracted samples of known concentration was performed. *n*-butanol alone had no effect upon the modified Kulka assay.

#### 2.3.6. Reagent Interference in the Modified Kulka Assay for DXP

Reagents at concentrations commonly used in DXS assays were tested for interference in the modified Kulka estimation of DXP in the DXS assay mix. (1) Dithiothreitol (DTT): 1 mM DTT decreased the A_564_ value to 70–90% of the control DXP values, with the number rising to 80 to 100% when DTT was used in the presence of 20-fold H_2_O_2_; (2) thiamine pyrophosphate (TPP): 0.1 mM TPP was not found to interfere with the assay. In the absence of DXP neither reagents contributed to the A_564_.

#### 2.3.7. Adaptation of the Modified Kulka Method for a High-Throughput Format

**Figure 6:**
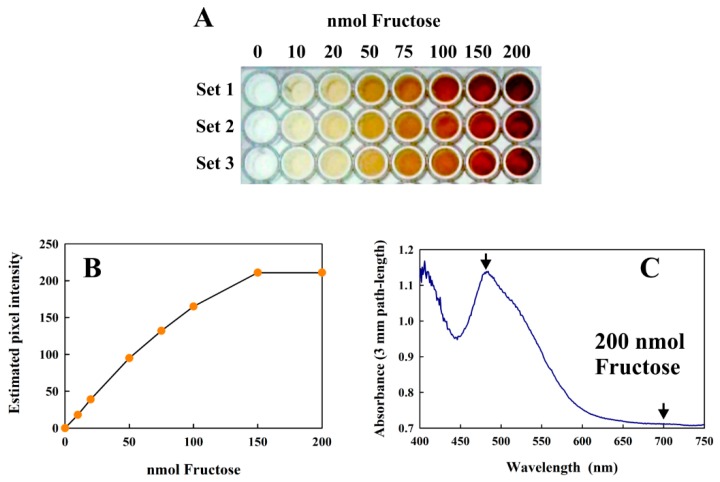
(**A**) The modified Kulka assay performed in 96-well plates using increasing amounts of fructose. Three replicates are shown (Sets 1–3) as a 24-bit color image. (**B**) Plot of the average pixel intensity from the center of each well in Set 1 of the RGB green channel 8-bit image (obtained from the 24-bit RGB image in (**A**)) vs. the amount of fructose present. (**C**) shows the absorption spectrum obtained from 100 μL of the Set 1 well (centrifuged) reaction mix containing 200 nmol fructose using a diode array spectrometer. The arrows indicate the measured absorbances.

The modified Kulka method can also be performed in 96-well plates, suitable for high-throughput applications. Since each well can only accommodate a total volume of 200 µL, all reagent volumes must be scaled down five-fold (see Materials and Methods for details). Although we have not performed a true high throughput assay in this study, we have successfully performed the reaction in a 96-well plates (Figure 6A) and indicated the results and limitations of different types of analysis (Figure 6B,C).

The calibration curve shown in Figure 6B shows that the assay can successfully be performed in this format and analyzed using the usual methods of quantitative image processing. However, at very high concentrations (above 150 nmol fructose), the images were too intense for precise analysis using this method. However, diode-array absorption spectra could be obtained reliably from even the highest values (Figure 6C), and the absorption difference between the visible peak maximum (at about 480 nm in this format) and the “baseline” at 700 nm corresponded well to the data shown in Figure 3 when corrections for the different path-lengths (conventional cuvettes, 1 cm; height of 100 µL sample in a 96-well plate: 3 mm) were made. The only significant limitation of the 96-well format was that the concentration of fructose in the 4 µL sample must be within the detection limits of the assay.

## 3. Discussion

In this study we have modified the well-known and very reliable Kulka assay method for the determination of furanoses [10] to use in modern high-throughput applications. Our modified method uses small volumes of reagents that can be handled safely with the usual pipettes and tips found in every biology laboratory. In our study we have only tested two furanoses: fructose, which finds many applications in biological contexts, and DXP, a substrate and product of two enzymes DXS, and DXP reductoisomerase [30,31], of the methylerythritol phosphate (MEP)-pathway of carotenoid biosynthesis in bacteria. In recent years, both of the latter enzymes have been found to be crucial for the production of precursors in biotechnological applications [32], so that a simple assay for them may often be convenient. DXP is also of interest in that it is a phosphorylated furanose, which has not been reported for use with the Kulka assay so far.

We have shown that the sensitivity and reliability for fructose determination with our modified assay corresponds well with that reported originally [10]. However, commonly observed turbidity effects have prompted us to use a two-wavelength measurement procedure for added reliability. We have also shown that the phosphorylated fructoses F6P and F1,6 diP were also measurable with the method. However, for the diphosphate, an appropriate calibration curve with the pure compound should be performed for additional precision.

We have also demonstrated that the assay was insensitive to the presence of small amounts of TCA, which is a convenient reagent for rapid deproteinization of samples. Indeed, TCA precipitation and removal of protein prior to resorcinol assay is recommended for biological samples. The assay was also insensitive to many of the salts and buffers commonly used in biological experiments, which has not been reported previously.

The assay proved to be very successful for the determination of DXP, even though the 1′-OH moiety, which is required for cyclic furfural product formation, was absent. However, the useful measurement range was limited for use in DXS assays, and required a concentration step prior to measurement. For this purpose, we have found sequential extraction with *n*-butanol to be effective, though this became tedious with large numbers of samples. In general, for the DXS assay, the modified Kulka method may be most convenient for a rapid assessment of the presence of enzyme activity. A more precise measurement will usually require a further determination by more advanced (usually HPLC) methods.

For workers wishing to determine other furanoses of interest, our work defines some initial tests that should be performed prior to employing the method. In particular, an absorption spectrum of the resorcinol-furanose adduct should be performed to assess the position of a characteristic absorption maximum, since the appropriate measurement wavelength can vary widely for different furanoses. Also, a displacement of the baseline of the absorption spectrum from that of the control (no reagent) can indicate significant turbidity, which should be treated as shown above (usually employing a two-wavelength measurement).

## 4. Materials and Methods

### 4.1. Chemicals

All chemicals were obtained from Sigma-Aldrich (Darmstadt, Germany) unless otherwise stated. The purple bacterium, *R. rubrum* S1 (ATCC no. 11170) was obtained from our in-house culture collection, and grown as described previously [22,23].

### 4.2. The Modified Kulka Protocol

Since the protocol requires an extended heating step at 80 °C, we routinely use 1.5 mL Safe-Lock^TM^ tubes (Eppendorf, Hamburg, Germany) for the method. For other 1.5 mL reaction tubes, the stability of the lid at 80 °C should be tested for a water-filled tube, and if necessary, every tube should be pierced with a 1.2 × 40 mm needle prior to heating. The latter will cause only very small losses due to evaporation while still ensuring lid stability.

We have reduced the number of reagents to three stock solutions: (1) Solution A or FAS-HCl reagent: 6.6 M HCl solution containing 0.9 mM Fe(III)(NH_4_)(SO_4_)_2_.12H_2_O; (2) Solution B: 1% (*w*/*v*) resorcinol (Sigma-Aldrich, Darmstadt, Germany) dissolved in neat ethanol; (3) Solution C: a fructose (or DXP) calibration aqueous solution. For fructose, we have found that fructose calibration solutions of 2.5 mM and 10 mM allowed for comfortable pipetting schemes for the ranges 0–100 nmol and 100–250 mmol fructose, respectively. Solution A remained stable in a tightly-closed bottle for at least two months at room temperature, the resorcinol solution had an even longer life (up to one year) at 4–6 °C, and fructose calibration solutions were stable for at least months as frozen aliquots at −20 °C. For the calibration with DXP, a 44 mM and a 2 mM stock solution in water was prepared and stored at −20 °C.

The modified Kulka protocol consisted of two steps: (a) color development of the resorcinol-furfural adduct: the appropriate amount (not more than 20 µL volume) of either Solution C or an unknown sample was added to 200 µL of solution A in a 1.5 mL SafeLock^TM^ tube, followed by the addition of 25 µL resorcinol reagent (solution B). The closed tube was then vortexed for 30 s, then incubated at 80 °C for 40 min, followed by a 3 min cooling period in ice. (b) The spectrophotometric step: to the cooled sample (at room temperature), 0.8 mL H_2_O was added to stop the reaction, mixed by vortexing, and then centrifuged for 5 min at 13,000 rpm (11,000× *g*) at room temperature. Subsequently, the supernatant was transferred to a fresh tube and the absorption measured at the wavelengths 470 nm (fructose) or 564 nm (DXP) and also at 700 nm (fructose only) using a 1 cm path-length cuvette. The calibration curve was calculated from the A_470_–A_700_ versus [fructose] or the A_564_ versus [DXP]. Since the DXP reaction product was not turbid, no centrifugation step was required for the DXP measurements and only a single measurement, A_564_, was sufficient for reliable determinations. For the initial part of the work, involving the assessment of the accuracy of the calibration assay, all measurements were performed in triplicate. Thereafter, for the model experiments described, only duplicate measurements were performed.

### 4.3. Deproteinization of Samples Prior to Fructose Determination

For samples containing significant amounts of protein, deproteinization of the sample prior to the assay with the modified Kulka method was performed as follows. TCA from a 72% (*w*/*v*) stock solution was added to the appropriate sample to yield a final concentration of 3.4% (*w*/*v*) (for samples containing only low amounts of protein), or 4.6% (*w*/*v*) (for analysis of cell cultures samples). After a 30 min incubation at room temperature, the sample was centrifuged in a desktop centrifuge for 20 min at 11,000× *g*, then the supernatant (containing the deproteinized fructose solution) was removed to a separate tube and immediately used to perform the assay using a 20 µL aliquot therefrom.

### 4.4. Absorption Spectroscopy

Routinely, absorption spectra (using 1 cm path-length quartz cuvettes (Starna scientific, Ilford, UK)) and also single absorption measurements (using plastic 1 cm path-length cuvettes (Roth, Karlsruhe, Germany)) were performed using a Jasco V-560 spectrophotometer (Jasco Corporation, Tokyo, Japan). However, 4 mm path-length plastic cuvettes were employed for turbidity measurements of *R. rubrum* cell cultures.

For the 100 µL reaction volumes in 96-well plates, absorption spectra were obtained in transmission mode using a J&M TIDAS diode array spectrometer (J&M Analytik AG, Essingen, Germany), using the procedure recommended by the manufacturer.

### 4.5. Simulation of a Progress Curve for DXS Enzyme Reaction

DXS catalyzes the TPP-dependent condensation of pyruvate and G3P to yield DXP. To simulate the progress curve, the integrated rate Equation (1) for a single-substrate irreversible reaction [33,34] was used:

(1) $$t=\frac{K_{m}}{V_{max}}*\left(ln\left(\frac{\left[S_{0}\right]}{\left[S_{0}\right]-\left[P\right]}\right)\right)+\frac{\left[P\right]}{V_{max}}$$

where *K_m_* and *V_max_* are the kinetic constants of the calculated reaction direction, [*S*_0_] is the initial concentration of one substrate *S*, and [*P*] is the calculated concentration of the product at a given time *t*.

Experimentally, this equation may be held valid when one of the substrates (e.g., pyruvate) is held at a saturating concentration, whereas the other substrate (e.g., G3P) is non-saturating. The kinetic constants for DXS as reported by Eubanks and Poulter [26] were used: *K_m_*^G3P^ = 0.068 mM, *K_m_*^pyruvate^ = 0.44 mM, and a *V_max_* (at saturating pyruvate concentration) = 0.45 µM/s was assumed throughout the reaction. The curve was calculated for the consumption of an initial [S_0_] or [G3P] of 0.5 mM together with concomitant, stoichiometric production of [DXP].

### 4.6. Procedures for Concentration of Sugar Phosphate Samples Using BaCl_2_ or n-Butanol

Aqueous samples of F6P (100 µL of 0.2–0.5 mM), dissolved in a 5 mM Na-phosphate buffer at pH 8.0, were precipitated with the addition of 10 µL 500 mM BaCl_2_ solution ([BaCl_2_]/[F6P] = 100) dissolved in 50 mM TrisHCl and an addition of 100 µL of 100% ethanol. For higher concentrations of F6P, a 10-fold molar excess of BaCl_2_ can be used for precipitation. The samples were incubated at 4 °C for 30 min, centrifuged at room temperature in a benchtop centrifuge at 11,000× *g* (13,000 rpm) for 5 min, and then the supernatant was removed. The assay was performed routinely in duplicates via the addition of FAS-HCl reagent, which immediately dissolves the Ba^2+^-F6P precipitate, and resorcinol was added to complete the assay cocktail.

To the aqueous DXP solution (100 µL aliquot, containing between 5–50 nmol DXP dissolved in H_2_O), 200 µL *n*-butanol was added, with pipettes and tips suitable for use with organic solvents and vortexed for 30 s and centrifuged at 11,000× *g* for 5 min at room temperature. The lower, aqueous layer was removed from the bottom of the tube and transferred to a fresh tube and extracted further with *n*-butanol as required. Two extraction steps with 200 µL *n*-butanol reduced the volume of the 100 µL DXP sample to approximately 25 µL. A smaller volume of *n*-butanol (25–100 µL) was used for finer reductions in the sample volume if required. Prior to the addition of the FAS-HCl reagent, samples were air dried inside a fume cupboard to remove traces of *n*-butanol. All dilute samples extracted by this method must be assayed in triplicates.

### 4.7. Adaptation of the Assay for High-Throughput Measurements Using 96-Well Plates

The assay can be adapted for 200 µL volumes, commonly used in plastic 96-well plates as follows. The initial reaction mixture in each well contains 40 µL FAS reagent and 5 µL resorcinol, to which 4 µL of fructose-containing sample was added. Since the sensitivity of the reaction remains unchanged in small volumes, the sample needed to be subjected to a concentration step prior to measurement. However, this requirement was also true for many other assays in metabolic studies, so we did not consider it explicitly here. The 96-well plate (with closed lid) was then incubated for 40 min at 80 °C, and then water was added to a final volume of 200 µL. The absorption measurement could then be performed using a plate reader or diode array spectrometer.

## Figures and Tables

**Table 1:** The effect of commonly used buffers and salts upon the modified Kulka assay.

Compound	Stock Soln ^a^	µmol in Assay ^b^	% of Control	Increase/Decrease
TrisHCl	100 mM	2	104	+
Na-Phosphate	100 mM	2	109	+
NaCl	100 mM	2	105	+
MgCl_2_	10 mM	0.2	105	+
HEPES ^d^	100 mM	2	107	+
MOPS ^d^	100 mM	2	104	+
TCA ^d^	72%	3.4–5% (*w*/*v*)	96–107	−
BSA ^d^	100 µg/mL	2 µg	120 ^c^	+
BSA/TCA	100 µg/mL	2 µg	99	−
Glucose	2.5 mM	20 nmol	103	+
Glucose only	2.5 mM	20 nmol	15	−
Glucose	111 mM	2.2 µmol	195	+
Glucose only	111 mM	2.2 µmol	142	+

^a^ The solution to be measured. Routinely, 20 µL of this solution was added to the resorcinol assay mix. ^b^ Unless otherwise stated, all assays contained 20 nmol of fructose. No fructose was present in the assays indicated as “Glucose only”. ^c^ Average value obtained with a BSA concentration of 100 µg/mL. ^d^ Abbreviations are given in the main text below.
